# Effect of Adverse Childhood Experiences on Nonsuicidal Self-Injury in Adolescents With Mood Disorders

**DOI:** 10.31083/AP52716

**Published:** 2026-10-14

**Authors:** Xinqi Wang, Fei Nie, Xi Zhang, Qiuping Tang, Chen Pan

**Affiliations:** ^1^Department of Clinical Psychology, The Third Xiangya Hospital, Central South University, 410013 Changsha, Hunan, China

**Keywords:** mood disorder, adverse childhood experiences, nonsuicidal self-injury, peer attachment, alexithymia

## Abstract

**Background:**

Nonsuicidal self-injury (NSSI) is a severe global health concern, particularly among adolescents, and is closely associated with adverse childhood experiences (ACEs). However, the underlying mechanisms by which ACEs are associated with NSSI remain largely unknown, although peer attachment and alexithymia have been identified as potentially relevant to this relationship. This observational study aimed to test a chain mediation model examining the indirect associations among ACEs, peer attachment, alexithymia, and NSSI in adolescents with mood disorders.

**Methods:**

A total of 363 adolescents with mood disorders were recruited from the outpatient clinical psychology department of a general hospital. Clinical interview tools and self-report questionnaires were used to evaluate patients’ history of NSSI, ACEs, peer attachment, and alexithymia. Comparisons were made between groups of patients who did and did not meet the criteria for NSSI diagnosis, and peer attachment and alexithymia were evaluated for potential mediating effects.

**Results:**

In total, 203 (55.9%) patients were included in the NSSI group. In the chain mediation model, ACEs were directly associated with NSSI, with a significant effect size of 0.246 (95% confidence interval [CI]: 0.088, 0.404), accounting for 64.2% of the total effect. Peer attachment and alexithymia had a total indirect effect of 0.137 (95% CI: 0.079, 0.221), accounting for 35.8% of the total effect.

**Conclusions:**

In addition to reaffirming the direct association between ACEs and NSSI, this study provides novel insight into a chain of indirect associations linking peer attachment and alexithymia to the ACE-NSSI relationship.

## Main Points

1. High prevalence: NSSI was identified in 55.9% of adolescents with mood disorders, with higher rates in females and younger patients.

2. Direct ACE–NSSI link: ACEs were significantly and positively associated with NSSI, serving as a key distal risk factor.

3. Alexithymia as core mediator: Alexithymia was the pivotal mediator—acting both independently and as a conduit for peer attachment’s indirect effect.

4. Chain mediation confirmed: ACEs indirectly influenced NSSI through the sequential pathway peer attachment → alexithymia; peer attachment alone did not mediate.

5. Clinical focus: Enhancing emotional recognition and expression may be a more direct and efficient intervention target for NSSI prevention and treatment.

## 1. Introduction

Nonsuicidal self-injury (NSSI) is a persistent and widely recognized public health concern throughout the globe. NSSI refers to any behavior that causes deliberate self-injury to the surface of the body without any suicidal intention [1,2]. NSSI prevalence rates are high, with an estimated lifetime prevalence of between 22% and 23% worldwide [3]. Of particular concern, these rates are rising, especially among adolescents [4]. The Diagnostic and Statistical Manual of Mental Disorders, Fifth Edition (DSM-5) acknowledges NSSI as a “condition requiring further study”. Rates of NSSI detection are higher among patients with clinical symptoms, with a particularly high prevalence in younger patients with mood disorders [5,6,7]. For example, NSSI was reported among 55.1% of depressed and 44.9% of bipolar adolescents in China [8]. NSSI is associated with several undesirable outcomes, including cognitive impairments, poor interpersonal relationships, and violent crime [9,10]. Given its prevalence and association with serious adverse consequences, there is a pressing need to investigate the NSSI profile of adolescent patients with mood disorders to identify risk factors associated with this behavior and inform the formulation of preventative and interventional strategies [11,12].

Findings from both clinical [13,14,15] and nonclinical studies [16,17], as well as a recent systematic review [18], provide support for adverse childhood experiences (ACEs) as an important risk factor associated with NSSI incidence. Experiencing severe childhood adversity has been tentatively linked with more frequent and severe NSSI events [19,20]. For example, in a cohort study by Russell et al. [21], a positive correlation was detected between ACE scores and the risk of self-injury among 16-year-olds, establishing ACEs as a significant distal risk factor for adolescent NSSI behaviors. Other studies have further delineated the relationship between experiences of childhood maltreatment and intentional self-harm, suggesting that multiple factors may mediate this association, including peer attachment and alexithymia [22,23].

ACEs can be devastating to the healthy development of an attachment system in children [24]. Insecure attachment can lead to a range of internalizing and externalizing problems [25], including a greater risk of NSSI [26]. Peer attachment forms a critical component of the attachment system through social support from friends, and has been shown to protect against self-injury in the context of ACEs [23,27]. Self-injury has been reported to be negatively associated with peer trust and communication as well as positively associated with peer alienation [28]. A longitudinal study by Martin et al. [29] demonstrated that ACEs such as abuse and neglect were associated with increases in NSSI frequency and severity, with attachment mediating this relationship. ACEs contribute to a greater risk of NSSI behaviors through their effects on an individual’s peer relationships, exacerbating individual challenges in the interpersonal domain during adolescence and thereby increasing the risk of NSSI behaviors [29].

Alexithymia is a condition characterized by difficulty in identifying and describing emotions [30]; it can be conceptualized as either a personality trait or a specific psychological reaction. Research has demonstrated a significant correlation between ACEs and alexithymia. Both childhood neglect and maltreatment are crucial factors in the development of alexithymia [31], and ACEs can contribute to the development of alexithymia in individuals with various psychopathological symptoms [22]. Several studies have shown a significant positive correlation between alexithymia and NSSI [32,33,34]. Individuals with high levels of alexithymia have difficulty understanding the information conveyed by others and are unable to provide timely and appropriate feedback; they tend to focus on negative aspects of the past and have a fatalistic interpretation of the present [35,36]. Thus, individuals with a history of ACEs tend to use maladaptive regulatory strategies, such as inhibition of expression and neglect, making them more vulnerable to engaging in NSSI behaviors. Alexithymia has recently been confirmed to mediate the relationship between ACEs and NSSI [37]. Moreover, several studies have demonstrated a correlation between alexithymia and insecure attachment relationships, indicating that adolescents’ interpersonal dynamics with their peers can influence their capacity to regulate their emotions [38,39].

According to attachment theory proposed by Bowlby [40], early interactions with caregivers shape internal working models of self and others, which form the foundation for personality development [41], influencing one’s eventual capacity for emotional regulation. Exposure to childhood maltreatment (ACEs) increases the risk of developing insecure attachment in childhood [42], and this insecurity often persists into adulthood [43,44,45], supporting the notion of attachment continuity. During adolescence, attachment extends from parents to peers, and peer attachment becomes a critical source of emotional support [46,47]. Insecure peer attachment has been linked to difficulties in the recognition and expression of emotions in the form of alexithymia. Specifically, caregivers who express emotions in maladaptive ways impair the ability of children to recognize and describe their emotions, fostering alexithymia [48,49,50]. Cerutti et al. [51] found that difficulties in emotional recognition and expression are significantly associated with attachment style, and that low attachment quality increases alexithymia, which in turn elevates the risk of NSSI. Peer attachment can thus be conceptualized as a distal interpersonal factor that influences alexithymia, while alexithymia serves as a proximal intrapersonal mechanism directly linked to NSSI.

Despite this theoretical and empirical grounding, few studies have simultaneously examined the sequential roles of peer attachment and alexithymia in the relationship between ACEs and NSSI. Moreover, most existing research has been conducted in nonclinical populations, limiting the generalizability of findings to clinical settings such as adolescents with mood disorders. Therefore, the present study aimed to examine the relationship between ACEs and NSSI in adolescents diagnosed with a mood disorder and to evaluate the influence of peer attachment and alexithymia on the association between ACEs and NSSI. Two hypotheses were formulated to guide this study: (a) ACEs are positively associated with NSSI, and (b) the association between ACEs and NSSI is indirectly mediated by peer attachment and alexithymia. The proposed relationships among these study variables are presented in Fig. 1.

**Fig. 1.**
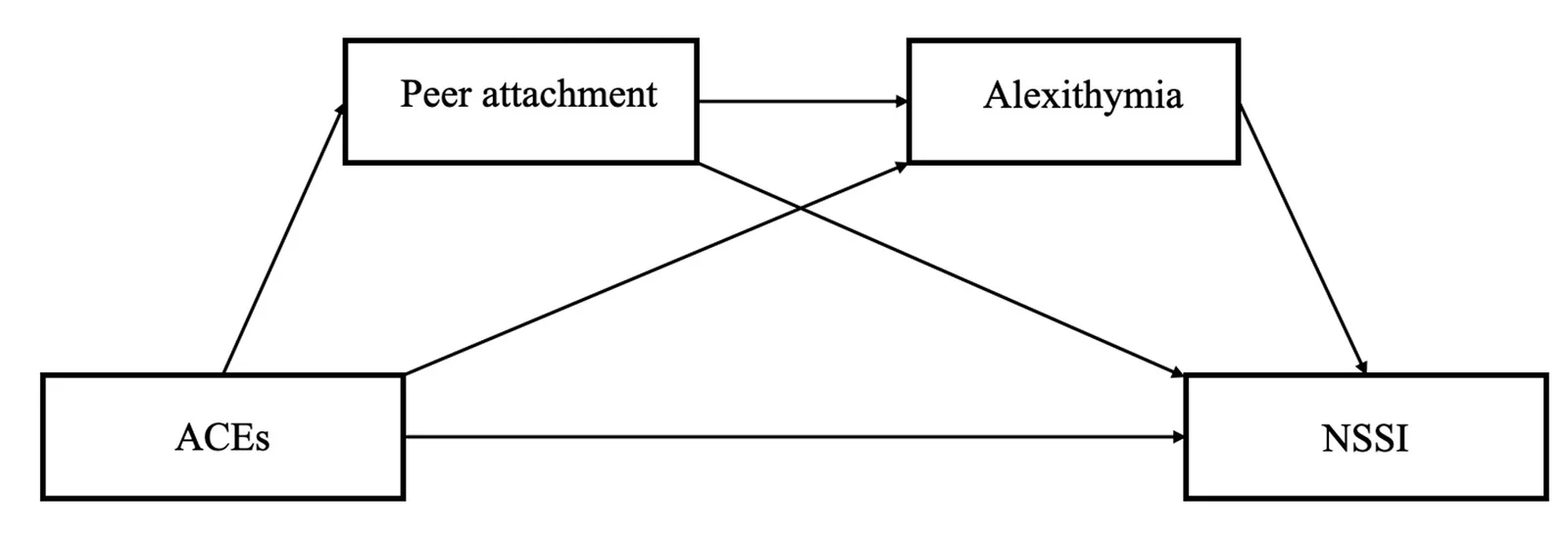
**Study framework**. Note: NSSI, nonsuicidal self-injury; ACEs, adverse childhood experiences.

## 2. Materials and Methods

### 2.1 Study Design and Participants

All study participants were recruited using convenience sampling from the outpatient clinic of the Department of Clinical Psychology at the Third Xiangya Hospital of Central South University in Hunan Province, China. The target sample size was calculated using G*Power (version 3.1, Heinrich-Heine-Universität Düsseldorf, Düsseldorf, North Rhine-Westphalia, Germany) [52], which indicated that 105 complete cases per study arm (210 total) would be necessary to detect a small between-group difference of Cohen’s *d *= 0.20 (α = 0.05; 90% power) on the primary outcome, and this sample size also satisfies the general requirement for mediation analysis. The data collection period spanned 11 months, from December 2022 to October 2023. The inclusion criteria were as follows: (a) age 12–20 years; (b) diagnosed with mood disorders, such as major depressive disorder, persistent depressive disorder, or bipolar mood disorder, per the appropriate DSM-5 diagnostic criteria; and (c) informed consent available from each participant and their parent(s)/caregivers. Participants with known structural cerebral/neurological comorbidities, manifestations of severe suicidal tendencies, anorexia, or catatonia, and those with intellectual disabilities were excluded from participation. All study procedures were performed in accordance with the Declaration of Helsinki.

### 2.2 Data Collection

All clinical diagnoses were made by two experienced psychiatrists (also the authors of this article). The data collection process comprised two phases: self-reported questionnaires followed by a clinical interview.

#### 2.2.1 Self-Reported Questionnaires

Information regarding sociodemographic variables, including gender, age, ethnicity, education levels, sibling status, family structure, and parental education, was obtained using a brief questionnaire.

ACEs were assessed using the Adverse Childhood Experiences International Questionnaire (ACE-IQ) [53]. For this study, we used the Chinese version of the ACE-IQ, which exhibits good reliability and validity in the Chinese population [54]. The ACE-IQ consists of 29 items covering 13 categories of ACEs assessed primarily across three dimensions: childhood abuse and neglect, family dysfunction, and extrafamilial violence. The total score ranged from 0 to 13, with higher scores being indicative of more ACEs. A frequency-based scoring method was employed in which an ACE had to reach a certain level of frequency before it was considered to have occurred.

Next, the peer attachment subscale of the Inventory of Parent and Peer Attachment (IPPA) scale [55] was used to assess levels of peer attachment among participating adolescents. The peer attachment subscale was employed to assess the current level of psychological security that individuals feel regarding their relationships with their peers. The subscale consists of 25 items covering three dimensions, including peer trust (10 items), peer communication (8 items), and peer alienation (7 items). The scale employs a 5-point Likert scale (1: “Almost Never”; 5: “Almost Always”), with reverse scoring for the peer alienation dimension. Higher scores on the scale indicate a better quality of peer attachment. The Chinese version of the IPPA has been frequently employed and exhibits high levels of reliability and validity among Chinese adolescents [56].

Lastly, the Chinese version of the 20-item Toronto Alexithymia Scale (TAS-20) was used to measure the level of alexithymia in study participants [57]. This scale evaluates three factors: difficulty discerning feelings (DDF, 7 items), difficulty expressing feelings (DEF, 5 items), and externally oriented thinking (EOT, 8 items). Items are rated using a 5-point Likert scale (1: “Completely Disagree”; 5: “Completely Agree”), where higher scores indicate a higher level of alexithymia. The scale exhibited good internal consistency (Cronbach’s α = 0.82) [57].

#### 2.2.2 Clinical Interviews

In this study, information related to NSSI was collected through clinical interviews conducted by trained psychology postgraduates in a separate interview room. The interview covered the following domains, corresponding to the full DSM-5 diagnostic criteria for NSSI (Section II, “Conditions for Further Study”): (a) Frequency of engaging in NSSI behavior in the past year (required: ≥5 days of intentional self‑inflicted damage to the body surface); (b) NSSI methods (hitting oneself, biting oneself, cutting or piercing the skin to draw blood, abrading skin to make it bleed, etc.) [58,59]; (c) Initial age of NSSI; (d) Causes of NSSI (the expectation that NSSI will solve an interpersonal problem, provide relief from unpleasant thoughts and/or emotions, or induce a positive emotional state); (e) NSSI-associated events (the presence of negative emotions, uncontrollable urge to self-harm, difficulties with interpersonal interactions). Patients were divided into two groups based on whether they met the diagnostic criteria for NSSI according to DSM-5, with those satisfying all of these criteria being classified into the NSSI group, while the remaining patients (including those with subthreshold NSSI, i.e., 1–4 days in the past year, as well as those with no NSSI) were categorized into the non-NSSI group.

### 2.3 Statistical Analysis

Descriptive statistics were calculated for both groups. Categorical data were expressed as frequency and percentages (%), whereas continuous data were expressed as mean ± standard deviation (SD). Age differences between the NSSI and non-NSSI groups were tested using an independent samples *t*-test, and categorical variables were analyzed using the chi-square test. Pearson’s correlation coefficients were used to analyze the relationships among these different factors. To examine the potential influence of common method bias, we performed Harman’s single-factor test. An unrotated principal component analysis on all 74 items from the ACE-IQ, the peer attachment subscale of the IPPA, and the TAS-20 revealed that the first factor accounted for 16.97% of the total variance, suggesting that common method bias is unlikely to be a serious problem in this study. When significant correlations were detected, indirect effect testing was performed with the SPSS Process 3.5 macro-plugin developed by Hayes [60]. Model 6 was proposed to analyze the mediation effects of peer attachment and alexithymia on ACEs and NSSI. ACEs were considered the independent variable, while NSSI was the dependent variable. All data analyses were conducted using SPSS 26.0 (SPSS Inc., Chicago, IL, USA), and a two-tailed *p *< 0.05 was considered significant.

## 3. Results

### 3.1 Prevalence of NSSI and Other Sociodemographic Characteristics

This study included 363 adolescent patients with mood disorders (mean age = 15.8 ± 2 years), of whom 252 (69.4%) were female and 111 (30.6%) were male. In total, 339 (93.4%) were Han Chinese. Medical records indicated that 37 of these patients (10.2%) were diagnosed with bipolar disorder (BD), 295 (81.3%) with major depressive disorder (MDD), and 31 (8.5%) with persistent depressive disorder (PDD). There were 203 (55.9%) patients in the NSSI group. In the non-NSSI group, 91 (34.6%) patients had no history of NSSI in the past year, whereas 69 (19%) patients had engaged in some NSSI behaviors in the past year but did not meet the diagnostic criteria for NSSI. Therefore, the non-NSSI group does not represent a control group without any history of self-injury. Instead, comparisons between the NSSI and non-NSSI groups reflect differences between those who meet diagnostic criteria for NSSI and those who do not. The sociodemographic and clinical characteristics of the study sample are presented in Table 1. Significant differences in age were evident between the two groups, with the NSSI group being younger (15.56 ± 1.98 years) than the non-NSSI group (16.29 ± 2.05 years; t(361) = –3.463, *p* = 0.001). Significant differences were also detected between the two groups in terms of gender (*p* = 0.003), father’s years of schooling (*p* = 0.037), diagnosis (*p* < 0.001), and medication use (*p* = 0.046); no significant differences were observed for any other variable.

**Table 1.** **Sociodemographic characteristics of NSSI and non-NSSI patients**.

Sociodemographic variables		Non-NSSI group (n = 160)		NSSI group (n = 203)		Total (N = 363)		Between-group differences
		n	%	n	%	n	%	*p*
Gender								0.003
	Female	98	61.3	154	75.9	252	69.4	
	Male	62	38.8	49	24.1	111	30.6	
Only child								0.584
	Yes	58	36.3	68	33.5	126	34.7	
	No	102	63.8	135	66.5	237	65.3	
Family structure								0.105
	Single parent	16	10.0	35	17.2	51	14.0	
	Both parents	141	88.1	162	79.8	303	83.4	
	Others	3	1.9	6	3.0	9	2.5	
Father’s education								0.037
	Primary and below	25	15.6	39	19.2	64	17.6	
	Junior High School	51	31.9	70	34.5	121	33.3	
	High School	29	18.1	51	25.1	80	22.0	
	College	24	15.0	15	7.4	39	10.7	
	Undergraduate	24	15.0	27	13.3	51	14.0	
	Postgraduate and above	7	4.4	1	0.5	8	2.2	
Mother’s education								0.112
	Primary and below	31	19.4	47	23.2	78	21.5	
	Junior High School	52	32.5	68	33.5	120	33.1	
	High School	28	17.5	44	21.7	72	19.8	
	College	29	18.1	26	12.8	55	15.2	
	Undergraduate	16	10.0	15	7.4	31	8.5	
	Postgraduate and above	4	2.5	3	1.5	7	1.9	
Diagnosis								<0.001
	Major depressive disorder	115	71.9	180	88.7	295	81.3	
	Bipolar mood disorder	20	12.5	17	8.4	37	10.2	
	Persistent depressive disorder	25	15.6	6	3.0	31	8.5	
Medication use								0.046
	Yes	34	21.3	62	30.5	96	26.4	
	No	126	78.7	141	69.5	267	73.6	

Note: NSSI, nonsuicidal self-injury. A two-tailed *p* < 0.05 was considered significant.

The onset age of NSSI in our study cohort ranged from 8 to 18 years (mean age = 13.92 ± 2.02 years). The most prevalent NSSI behaviors were hitting oneself (90.1%), biting oneself (68.5%), and cutting or piercing the skin to draw blood (66.5%). The primary motivation for NSSI was to alleviate negative thoughts or emotions (90.6%), followed by seeking positive feelings (30%), and resolving relationship issues (17.2%). The most frequently reported triggers for NSSI were the presence of negative emotions (92.6%), followed by an uncontrollable urge to self-harm (58.1%) and difficulties with interpersonal interactions (47.3%). For further details, see Table 2.

**Table 2.** **NSSI modalities, causes, and associated events for patients in the NSSI group**.

Variable	Items	n	%
NSSI methods	Hitting oneself	183	90.1
	Biting oneself	139	68.5
	Cutting or piercing the skin to draw blood	135	66.5
	Abrading skin to make it bleed	66	32.5
	Inserting objects under the skin or nails	24	11.8
	Burning the skin	17	8.4
Primary motivation	Alleviate negative thoughts or emotions	184	90.6
	Seeking positive feelings	61	30
	Resolving relationship issues	35	17.2
	Others	9	4.4
Triggers for NSSI	The presence of negative emotions	188	92.6
	Uncontrollable urge to self-harm	118	58.1
	Difficulties with interpersonal interactions	96	47.3
	Others	4	2.0

Note: NSSI, nonsuicidal self-injury.

### 3.2 ACEs and NSSI

Of the 363 patients with adolescent mood disorders, 325 (89.5%) reported ACEs; of these, 250 (68.9%) patients reported multiple instances of ACEs. Significant differences between the NSSI and non-NSSI groups were detected in terms of emotional neglect (χ^2^ = 29.569; *p* < 0.001), physical neglect (χ^2^ = 3.996; *p* = 0.046), parental divorce or death (χ^2^ = 5.042; *p* = 0.025), violent treatment of family members (χ^2^ = 3.995; *p* = 0.046), emotional abuse (χ^2^ = 4.904;* p* = 0.027), physical abuse (χ^2^ = 6.994; *p* = 0.008), sexual abuse (χ^2^ = 5.629; *p* = 0.018), and community violence (χ^2^ = 14.057; *p* < 0.001). No significant differences were noted in other factors between the two groups (Table 3).

**Table 3.** **ACE comparisons between adolescents in the NSSI and non-NSSI groups**.

Variable			Non-NSSI group (n = 160)		NSSI group (n = 203)		Total (N = 363)		Group differences	
			N	%	N	%	N	%	χ^2^	*p*
ACEs										
	0		27	16.9	11	5.4	38	10.5	12.530	<0.001
	≥1		133	83.1	192	94.6	325	89.5		
Abuse and neglect										
	Emotional neglect									
		Yes	95	59.4	172	84.7	267	73.6	29.569	<0.001
		No	65	40.6	31	15.3	96	26.4		
	Physical neglect									
		Yes	0	0	5	2.5	5	1.4	3.996	0.046
		No	160	100	198	97.5	358	98.6		
	Emotional abuse									
		Yes	29	18.1	57	28.1	86	23.7	4.904	0.027
		No	131	81.9	146	71.9	277	76.3		
	Physical abuse									
		Yes	6	3.8	23	11.3	29	8.0	6.994	0.008
		No	154	96.4	180	88.7	334	92.0		
	Sexual abuse									
		Yes	22	13.8	48	23.6	70	19.3	5.629	0.018
		No	138	86.3	155	76.4	293	80.7		
Family dysfunction										
	Family member’s substance abuse									
		Yes	9	5.6	23	11.3	32	8.8	3.623	0.057
		No	151	94.4	180	88.7	331	91.2		
	Family member’s mental illness or suicide									
		Yes	11	6.9	15	7.4	26	7.2	0.036	0.850
		No	149	93.1	188	92.6	337	92.8		
	Family member imprisoned									
		Yes	7	4.4	15	7.4	22	6.1	1.428	0.232
		No	153	95.6	188	92.6	341	93.9		
	Divorce or death of parents									
		Yes	35	21.9	66	32.5	101	27.8	5.042	0.025
		No	125	78.1	137	67.5	262	72.2		
	Violent treatment of family member									
		Yes	25	15.6	49	24.1	74	20.4	3.995	0.046
		No	135	84.4	154	75.9	289	79.6		
Extra-family violence										
	Peer violence									
		Yes	11	6.9	22	10.8	33	9.1	1.700	0.192
		No	149	93.1	181	89.2	330	90.9		
	Community violence									
		Yes	0	0.0	17	8.4	17	4.7	14.057	<0.001
		No	160	100	186	91.6	346	95.3		
	Collective violence									
		Yes	10	6.3	23	11.3	33	9.1	2.794	0.095
		No	150	93.8	180	88.7	330	90.9		

Note: Patient ACE-related information was collected using the Adverse Childhood Experiences International Questionnaire (ACE-IQ). NSSI, nonsuicidal self-injury; ACEs, adverse childhood experiences. A two-tailed *p*-value < 0.05 was considered significant.

### 3.3 Peer Attachment and Alexithymia

Table 4 provides a summary of variables associated with peer attachment and alexithymia among study participants. Overall, the NSSI group exhibited lower levels of peer attachment and higher levels of alexithymia as compared to the non-NSSI group (t = –4.203, *p* < 0.001; t = 8.373, *p* < 0.001, respectively).

**Table 4.** **Peer attachment and alexithymia in the NSSI and non-NSSI groups**.

Variable		Non-NSSI group (n = 160)	NSSI group (n = 203)	Total (N = 363)	Group differences	
		Mean ± SD	Mean ± SD	Mean ± SD	t	*p*
Peer attachment		81.08 ± 14.57	74.27 ± 15.87	77.27 ± 15.66	–4.203	<0.001
	Peer communication	25.76 ± 5.63	23.68 ± 6.50	24.60 ± 6.21	–3.252	0.001
	Peer alienation	21.41 ± 4.05	19.25 ± 4.85	20.20 ± 4.63	–4.555	<0.001
	Peer trust	33.90 ± 6.84	31.34 ± 8.07	32.46 ± 7.70	–3.206	0.001
Alexithymia		61.76 ± 10.82	70.19 ± 8.35	66.47 ± 10.39	8.373	<0.001
	DDF	23.40 ± 5.87	27.93 ± 4.49	25.93 ± 5.61	8.325	<0.001
	DEF	16.59 ± 3.76	19.03 ± 2.88	17.96 ± 3.51	6.994	<0.001
	EOT	21.77 ± 4.36	23.23 ± 4.13	22.59 ± 4.29	3.268	0.001

Note: NSSI, nonsuicidal self-injury; DDF, difficulty discerning feelings; DEF, difficulty expressing feelings; EOT, externally oriented thinking; A two-tailed *p*-value < 0.05 was considered significant.

### 3.4 Correlations Between Main Variables

Bivariate correlations between variables were next tested with Pearson’s correlation analysis. To control for multiple comparisons, the Benjamini‑Hochberg false discovery rate (FDR) procedure was applied to all correlation coefficients, with q = 0.05. After FDR correction, all reported correlations remained statistically significant. As shown in Table 5, NSSI was positively associated with ACEs (r = 0.286; *p* < 0.001) and alexithymia (r = 0.403; *p* < 0.001), whereas it was negatively associated with peer attachment (r = –0.216; *p* < 0.001). ACEs were negatively associated with peer attachment (r = –0.268; *p* < 0.001) and positively associated with alexithymia (r = 0.341;* p* < 0.001).

**Table 5.** **Evaluation of correlations among NSSI, ACEs, peer attachment, and alexithymia (N = 363)**.

Variable	NSSI	ACEs	Peer attachment	Peer trust	Peer communication	Peer alienation	Alexithymia	DDF	DEF	EOT
NSSI	1.000									
ACEs	0.286**	1.000								
Peer attachment	–0.216**	–0.268**	1.000							
Peer trust	–0.166**	–0.225**	0.949**	1.000						
Peer communication	–0.166**	–0.199**	0.909**	0.878**	1.000					
Peer alienation	–0.233**	–0.270**	0.595**	0.381**	0.285**	1.000				
Alexithymia	0.403**	0.341**	–0.475**	–0.409**	–0.393**	–0.404**	1.000			
DDF	0.401**	0.323**	–0.373**	–0.304**	–0.264**	–0.406**	0.873**	1.000		
DEF	0.345**	0.235**	–0.420**	–0.339**	–0.363**	–0.372**	0.799**	0.678**	1.000	
EOT	0.169**	0.211**	–0.319**	–0.316**	–0.310**	–0.143*	0.627**	0.253**	0.231**	1.000

Note: NSSI, nonsuicidal self-injury; ACEs, adverse childhood experiences; DDF, difficulty discerning feelings; DEF, difficulty expressing feelings; EOT, externally oriented thinking. **p < 0.01; *p < 0.05. All correlation coefficients remained significant after Benjamini-Hochberg false discovery rate correction (q = 0.05).

### 3.5 Peer Attachment and Alexithymia as Mediators of the Relationship Between ACEs and NSSI

Before testing the mediation model, a linear regression approach was employed to examine the associations between variables using linear regression for continuous outcomes (peer attachment and alexithymia) and binary logistic regression for NSSI. Covariates incorporated into each model included gender, age, father’s education, diagnostic subtype, and medication use. The results of the linear regression analysis revealed that ACEs were significantly associated with peer attachment (B = –2.031, *p* < 0.001) and alexithymia (B = 1.165, *p* < 0.001), while peer attachment was also significantly associated with alexithymia (B = –0.250, *p* < 0.001) (Table 6). The results of the logistic regression analysis further indicated that ACEs (B = 0.246, *p* = 0.002) and alexithymia (B = 0.078, *p* < 0.001) were significant positive predictors of NSSI group membership, whereas peer attachment was not (B = –0.003, *p* = 0.715) (Table 7). These results only present the coefficients for the key predictors (ACEs, peer attachment, and alexithymia); for the full regression tables, see the **Supplementary Tables 1 and 2**.

**Table 6.** **Linear regression results for peer attachment and alexithymia**.

Outcome variable	Predictor variable	B	SE	t	*p*	95% CI for B
Peer attachment	ACEs	–2.031	0.421	–4.825	<0.001	[–2.859, –1.203]
Alexithymia	ACEs	1.165	0.253	4.597	<0.001	[0.665, 1.661]
	Peer attachment	–0.250	0.031	–8.137	<0.001	[–0.312, –0.190]

Note: ACEs, adverse childhood experiences. B, unstandardized coefficient; SE, standard error; CI, confidence interval. Covariates incorporated into all linear regression models included gender, age, father’s education, diagnostic subtype, and medication use.

**Table 7.** **Binary logistic regression results for NSSI**.

Predictor	B	SE	Z	*p*	95% CI for B	OR (Exp(B))	95% CI for OR
ACEs	0.246	0.081	3.046	0.002	[0.088, 0.404]	1.279	[1.092, 1.498]
Peer attachment	–0.003	0.009	–0.366	0.715	[–0.022, 0.015]	0.997	[0.978, 1.015]
Alexithymia	0.078	0.016	4.926	<0.001	[0.047, 0.109]	1.081	[1.048, 1.115]

Note: B, unstandardized log-odds coefficient; SE, standard error; Z = B/SE; OR, odds ratio. Nagelkerke R^2^ = 0.341 (from the logistic regression model). All covariates were entered simultaneously.

The hypothetical chain mediation effect of peer attachment and alexithymia was verified using the SPSS Process macro plug-in model 6 (chain mediation model). The hypothesized model was tested, and the mediation effect values were calculated along with 95% confidence intervals (CIs) using 5000 bootstrap samples, adjusting for gender, age, father’s education, diagnostic subtype, and medication use. The proposed pathway through which ACEs influence NSSI via peer attachment and alexithymia is presented in Fig. 2.

**Fig. 2.**
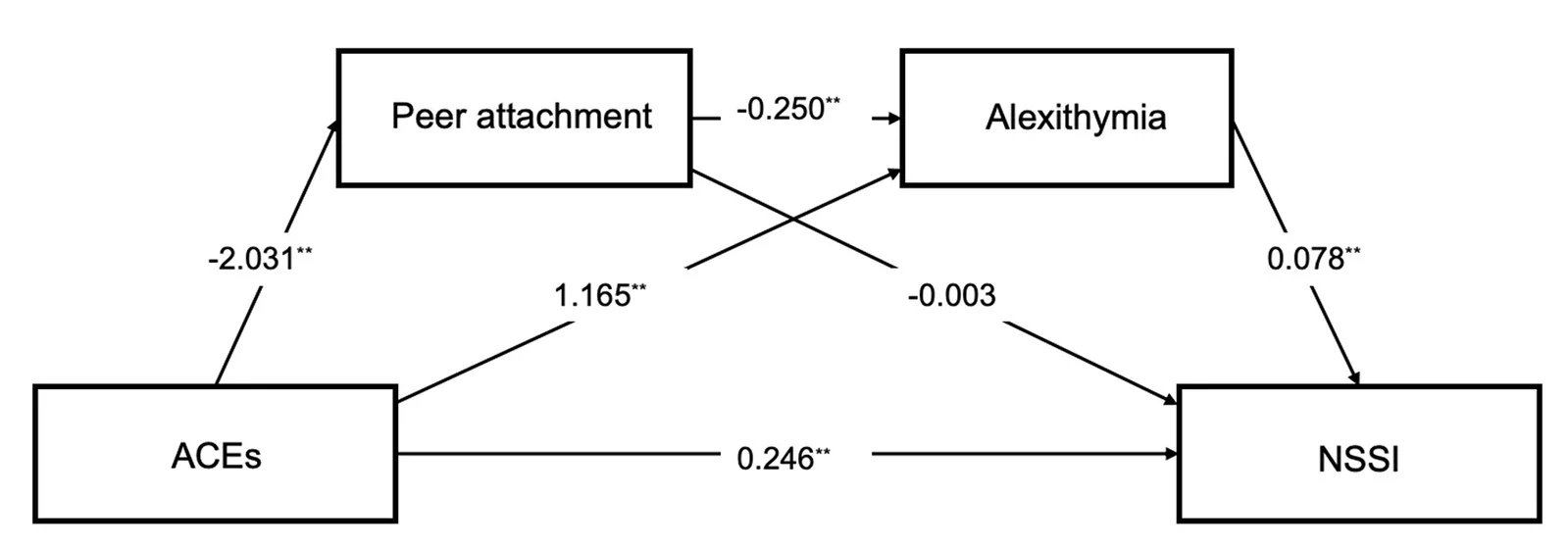
**Chain mediation model: indirect statistical associations of peer attachment and alexithymia linking ACEs to NSSI**. Note: NSSI, nonsuicidal self-injury; ACEs, adverse childhood experiences. **p < 0.01.

These results highlighted a direct statistical association between ACEs and NSSI, with a coefficient of 0.246 (95% CI: 0.088, 0.404), accounting for 64.2% of the total association. Additionally, the total indirect statistical association via peer attachment and alexithymia was 0.137 (95% CI: 0.079, 0.221), accounting for 35.8% of the total association. When evaluating specific paths within this model, Path 1 did not show significance, whereas Path 2 exhibited a significant effect value of 0.090, accounting for 65.7% of the total indirect effect. Path 3 was also significant, with an effect value of 0.040, accounting for 29.2% of the total indirect effect.

As the indirect path mediated via peer attachment alone (Path 1) was not significant, this indicates that peer attachment does not independently mediate the relationship between ACEs and NSSI. In contrast, the significant indirect paths were those involving alexithymia (Path 2 and Path 3). This pattern suggests that alexithymia is the key mediating factor, while peer attachment may function only as a distal factor that influences alexithymia, which in turn is associated with NSSI. These findings suggest a direct statistical association between ACEs and NSSI, as well as an indirect statistical association via the chain of peer attachment and alexithymia. Table 8 shows the results of this chain mediation analysis.

**Table 8.** **Mediation effect analysis of peer attachment and alexithymia on NSSI**.

	Effect	Boot SE	LLCI	ULCI
Direct effect	0.246	0.081	0.088	0.404
Indirect effect	0.137	0.036	0.079	0.221
Ind1 ACEs → peer attachment → NSSI	0.007	0.021	–0.033	0.051
Ind2 ACEs → alexithymia → NSSI	0.090	0.027	0.045	0.151
Ind3 ACEs → peer attachment → alexithymia → NSSI	0.040	0.016	0.020	0.076

Note: NSSI, nonsuicidal self-injury; ACEs, adverse childhood experiences; LLCI, lower limit of confidence interval; ULCI, upper limit of confidence interval; Ind1, Ind2, and Ind3 are the three possible paths in the mediated regulation model.

## 4. Discussion

This study examined NSSI frequencies and patterns in a clinical cohort of adolescent patients diagnosed with mood disorders. In addition, we investigated the associations between NSSI and ACEs, peer attachment, and alexithymia, while also exploring how ACEs contribute to the occurrence of NSSI. Through these analyses, we were able to replicate and extend previously reported findings in addition to providing data to support the role of peer attachment and alexithymia as chained mediators of ACEs and NSSI in this population.

### 4.1 Key Features of NSSI in Adolescents With Mood Disorders

In line with previous research, NSSI was prevalent among individuals with mood disorders in our own clinical cohort [61,62], particularly among female patients [63]. Two potential explanations for gender-related differences in adolescent NSSI have been proposed: physiological factors, such as the differing levels of hormones, and social differences in the strategies employed for emotional regulation. Previous studies have noted certain gender-related differences in ability and style with respect to the regulation of negative emotions. Males tend to employ cognitive reappraisal, whereas females are more reliant on rumination, such that they are more prone to NSSI [64].

We also detected a significant difference between the NSSI and non-NSSI groups in terms of the fathers’ educational levels. Many studies have focused on the impact of paternal education on health outcomes among children and found that paternal education is positively associated with self-rated health [65], such that children of more educated fathers exhibit better health outcomes than their peers with less educated fathers [66]. It is important to note, however, that the impact of paternal education on children’s health outcomes varies considerably between Eastern and Western contexts [65]. Specifically, the traditional concept of the “patriarchal system” still has a profound impact in China, where the father has an important role, status, and responsibility in the family that is often significantly associated with children’s growth and development.

### 4.2 ACEs as A Risk Factor for NSSI

The present study detected a notable direct association between ACEs and NSSI, consistent with previous research identifying ACEs as a risk factor for NSSI [67,68]. The more ACEs, such as parental neglect, abuse, or deprivation, one has experienced, the greater the likelihood of the child engaging in NSSI [69].

ACEs increase vulnerability to NSSI. ACEs have been shown to be associated with myriad mental- and physical health problems throughout the lifespan [70,71]. ACEs can be “devastating” to an individual’s development, affecting the evolution of a wide range of adaptive skills. For example, individuals with ACEs are significantly more emotionally reactive when confronted with stimuli that may signal a threat or danger [71]. Those who grow up in adverse environments retain the coping styles (e.g., isolation, somatization) and defenses (e.g., self-punishment) they had in infancy and early childhood [72]. However, with changing physical, psychological, and social environments, these primitive skills lose their adaptive utility. With stress accumulation, maladaptive behaviors like NSSI may emerge as an effective tool for regulating emotions, protecting the integrity of the self, and gaining a sense of connection to reality.

### 4.3 The Mediating Role of Peer Attachment and Alexithymia

In addition to finding a direct statistical association between ACEs and NSSI, we identified significant indirect pathways mediating this relationship: ACEs-alexithymia-NSSI and ACEs-peer attachment-alexithymia-NSSI. Notably, the indirect pathway via peer attachment alone (ACEs-peer attachment-NSSI) was not significant (see Table 8, Ind1). This indicates that peer attachment does not independently mediate the relationship between ACEs and NSSI. Instead, alexithymia is the key mediating factor, and peer attachment may function only as a distal factor that influences alexithymia. These findings are consistent with the observation that individuals with more ACEs are more likely to report poorer peer attachment, higher levels of alexithymia, and a greater likelihood of NSSI behaviors.

In line with prior work, we found that ACEs tended to co-occur with the development of insecure attachment patterns [73]. Both ACEs and this insecure attachment pattern cause individuals to develop an experiential avoidance of emotional expression, most often in the form of repression and rejection [32], and even a sense of discomfort with affirmation from others that is difficult to fully understand and describe [74]. In this context, the distress associated with difficulties in emotional regulation may be linked to attempts to shift attention from internal feelings to the external body through risky but perceived “beneficial” means such as NSSI [75], and to acquire the ability to recognize and describe emotions [76]. For example, NSSI behaviors in individuals with difficulties in emotional discrimination may be motivated by emotional regulation, and NSSI in individuals with difficulties in describing emotions may be motivated by communication [34].

Although the chain of indirect associations involving peer attachment and alexithymia was significant in our analysis, no corresponding significance was noted for the separate indirect association via peer attachment alone, whereas alexithymia alone showed a significant indirect association. This finding appears inconsistent with some previous studies in which peer attachment was identified as an independent mediator in community samples [77]. A likely explanation for this discrepancy lies in the clinical nature of our sample. Specifically, adolescents with diagnosed mood disorders experience severe emotional distress and psychopathology, which may overshadow the direct protective effect of peer attachment, making alexithymia a more dominant mechanism. Those individuals with a history of ACEs may develop negative relationships with their peers. Nevertheless, peer attachment does not directly account for NSSI; rather, it contributes to NSSI indirectly through alexithymia. This suggests that intra-individual factors (alexithymia) may be more strongly associated with NSSI than socio-environmental factors (peer attachment) in this population [78]. This finding may have implications for clinical intervention. Efforts to improve an individual’s capacity for emotional regulation (e.g., by training the ability to discriminate and communicate emotions) may therefore be a promising approach for clinicians seeking to manage patients with NSSI behaviors. This ability represents the most direct and effective means of preventing individuals from engaging in self-injurious behaviors. In addition, it is more malleable than the social environment in which the individual lives and offers the promise of intervention in a relatively short period of time. Even so, it is important for clinical therapists to consider the social environment of NSSI patients, as a supportive social environment may facilitate the acquisition of adaptive skills, even if it does not directly cause NSSI.

### 4.4 Limitations and Prospects for the Future

This study offers valuable insight into the association between ACEs and NSSI, as well as the roles of peer attachment and alexithymia in mediating this relationship. However, several limitations should be acknowledged.

Firstly, the present study employed a cross-sectional design, which may not have fully captured the dynamic nature of self-injurious behaviors in adolescents with mood disorders. Moreover, cross-sectional data cannot establish temporal precedence or causal direction, even when testing a theoretically driven chain model. Therefore, these findings should be interpreted as associations consistent with the proposed model, rather than as proof of causation. Future longitudinal studies are needed to test the temporal ordering of the variables and to better account for the dynamics of NSSI. Secondly, we did not employ standardized mood symptom rating scales to quantify the severity of depression or mania in our cohort. Consequently, we could not adjust for dimensional measures of symptom severity. Future studies should include such measures to further rule out any confounding effects linked to clinical severity. Other limitations include our reliance on single-center convenience sampling, self‑report measures, and the use of a total ACE score rather than subtype-specific analyses. As such, future studies should employ larger samples to examine disorder-specific pathways, include medication use as a covariate, and further explore more fine-grained diagnostic subtypes to improve generalizability.

## 5. Conclusions

In conclusion, the findings of this study indicate that NSSI is a prevalent phenomenon among adolescents with mood disorders, particularly among girls and adolescents with low levels of paternal education. ACEs were found to be directly associated with NSSI, in addition to being indirectly associated with these injurious behaviors via peer attachment and alexithymia. Specifically, the association between ACEs and NSSI was statistically mediated by alexithymia alone and by the chain of peer attachment followed by alexithymia. These results indicate that interventions designed to enhance the ability of adolescents with mood disorders to recognize and express emotions may be of significant benefit.

## Data Availability

The datasets generated and analyzed during the current study are not publicly available due to privacy or ethical restrictions but are available from the corresponding author on reasonable request.

## Abbreviations

NSSI, Nonsuicidal Self-Injury; ACEs, Adverse Childhood Experiences; DSM-5, The Diagnostic and Statistical Manual of Mental Disorders, Fifth Edition; ACE-IQ, Adverse Childhood Experiences International Questionnaire; IPPA, Inventory of Parent and Peer Attachment; TAS-20, The 20-item Toronto Alexithymia Scale; DDF, Difficulty Discerning Feelings; DEF, Difficulty Expressing Feelings; EOT, Externally Oriented Thinking; SD, Standard Deviation; CI, Confidence Intervals; LLCI, Lower Limit of Confidence Interval; ULCI, Upper Limit of Confidence Interval.
