# Supplementary material for: Effect of Adverse Childhood Experiences on Nonsuicidal Self-Injury in Adolescents With Mood Disorders
Source: Alpha Psychiatry. 2026 Oct 14;27(5):52716. doi: 10.31083/AP52716 (PMC13731992; doi:10.31083/AP52716)
Supplement: Supplementary file 1 [file 2757-8038-27-5-52716-s1.zip › Supplementary Material.docx]

**Supplement Material**

**Supplementary Table 1. Linear regression results for peer attachment and alexithymia.**

| Outcome variable | Predictor variable | B | SE | t | *p* | *95% CI for B* |
| --- | --- | --- | --- | --- | --- | --- |
| Peer attachment | ACEs | -2.031 | 0.421 | -4.825 | <0.001 | [-2.859, -1.203] |
|  | Gender | 0.600 | 1.707 | 0.351 | 0.726 | [-2.757, 3.956] |
|  | Age | 0.660 | 0.386 | 1.709 | 0.088 | [-0.100, 1.420] |
|  | Father’s education | 1.502 | 0.552 | 2.722 | 0.007 | [0.417, 2.587] |
|  | Diagnostic subtype | 1.507 | 1.308 | 1.152 | 0.250 | [-1.065, 4.079] |
|  | Medication use | 4.279 | 1.795 | 2.384 | 0.018 | [0.749, 7.809] |
| Alexithymia | ACEs | 1.163 | 0.253 | 4.597 | <0.001 | [0.665, 1.661] |
|  | Peer attachment | -0.251 | 0.031 | -8.137 | <0.001 | [-0.312, -0.190] |
|  | Gender | 1.187 | 0.994 | 1.194 | 0.233 | [-0.768, 3.143] |
|  | Age | -0.586 | 0.227 | -2.585 | 0.010 | [-1.032, -0.140] |
|  | Father’s education | -0.545 | 0.333 | -1.636 | 0.103 | [-1.200, 0.110] |
|  | Diagnostic subtype | -1.889 | 0.778 | -2.427 | 0.016 | [-3.420, -0.359] |
|  | Medication use | -0.883 | 1.064 | -0.830 | 0.407 | [-2.976, 1.209] |

Note. ACEs, adverse childhood experiences. B, unstandardized coefficient; SE, standard error; CI, confidence interval.

**Supplementary Table 2. Binary logistic regression results for NSSI.**

| Predictor | B | SE | Z | p | 95% CI for B | OR (Exp(B)) | 95% CI for OR |
| --- | --- | --- | --- | --- | --- | --- | --- |
| ACEs | 0.246 | 0.081 | 3.046 | 0.002 | [0.088, 0.404] | 1.279 | [1.092, 1.498] |
| Peer attachment | -0.003 | 0.009 | -0.366 | 0.715 | [-0.022, 0.015] | 0.997 | [0.978, 1.015] |
| Alexithymia | 0.078 | 0.016 | 4.926 | <0.001 | [0.047, 0.109] | 1.081 | [1.048, 1.115] |
| Gender | 0.708 | 0.271 | 2.611 | 0.009 | [0.176, 1.239] | 2.029 | [1.193, 3.452] |
| Age | -0.106 | 0.062 | -1.703 | 0.089 | [-0.227, 0.016] | 0.900 | [0.797, 1.016] |
| Father’s education | -0.071 | 0.088 | -0.812 | 0.417 | [-0.244, 0.101] | 0.931 | [0.783, 1.106] |
| Diagnostic subtype | -0.616 | 0.221 | -2.786 | 0.005 | [-1.050, -0.183] | 0.540 | [0.350, 0.833] |
| Medication use | 0.835 | 0.301 | 2.779 | 0.005 | [0.246, 1.424] | 2.305 | [1.279, 4.155] |
| Constant | -3.925 | 1.908 | -2.057 | 0.040 | [-7.665, -0.185] | 0.020 | – |

Note. B, unstandardized log-odds coefficient; SE, standard error; Z = B/SE; OR, odds ratio. Nagelkerke R² = 0.341(from the logistic regression model). All covariates were entered simultaneously.
